# HPV16 E6 Controls the Gap Junction Protein Cx43 in Cervical Tumour Cells

**DOI:** 10.3390/v7102871

**Published:** 2015-10-05

**Authors:** Peng Sun, Li Dong, Alasdair I. MacDonald, Shahrzad Akbari, Michael Edward, Malcolm B. Hodgins, Scott R. Johnstone, Sheila V. Graham

**Affiliations:** 1 Feinberg School of Medicine, North Western University, Chicago, IL 60611, USA; pengsun2014@gmail.com; 2 MRC-University of Glasgow Centre for Virus Research, Institute of Infection, Immunity and Inflammation, College of Medical, Veterinary and Life Sciences, University of Glasgow, Garscube Estate, Glasgow G61 1QH, Scotland, UK; 2102286D@student.gla.ac.uk (L.D.); Alasdair.MacDonald@gla.ac.uk (A.I.M.); 2107107A@student.gla.ac.uk (S.A.); 3 Dermatology, School of Medicine, College of Medical, Veterinary and Life Sciences, University of Glasgow, Glasgow G12 8TT, Scotland, UK; Mike.Edward@gla.ac.uk (M.E.); Malcolm.Hodgins@gla.ac.uk (M.B.H.); 4 Institute of Cardiovascular and Medical Sciences, College of Medical, Veterinary and Life Sciences, University of Glasgow, Glasgow G12 8TT, Scotland, UK; Scott.Johnstone@gla.ac.uk

**Keywords:** human papillomavirus (HPV), E6 oncoprotein, Connexin 43 (Cx43) trafficking, human homologue of *Drosophila* discs large (hDlg)

## Abstract

Human papillomavirus type 16 (HPV16) causes a range of cancers including cervical and head and neck cancers. HPV E6 oncoprotein binds the cell polarity regulator hDlg (human homologue of *Drosophila* Discs Large). Previously we showed *in vitro*, and now *in vivo*, that hDlg also binds Connexin 43 (Cx43), a major component of gap junctions that mediate intercellular transfer of small molecules. In HPV16-positive non-tumour cervical epithelial cells (W12G) Cx43 localised to the plasma membrane, while in W12T tumour cells derived from these, it relocated with hDlg into the cytoplasm. We now provide evidence that E6 regulates this cytoplasmic pool of Cx43. E6 siRNA depletion in W12T cells resulted in restoration of Cx43 and hDlg trafficking to the cell membrane. In C33a HPV-negative cervical tumour cells expressing HPV16 or 18 E6, Cx43 was located primarily in the cytoplasm, but mutation of the 18E6 C-terminal hDlg binding motif resulted in redistribution of Cx43 to the membrane. The data indicate for the first time that increased cytoplasmic E6 levels associated with malignant progression alter Cx43 trafficking and recycling to the membrane and the E6/hDlg interaction may be involved. This suggests a novel E6-associated mechanism for changes in Cx43 trafficking in cervical tumour cells.

## 1. Introduction

Gap junctions are specialized cell membrane channels that allow direct intercellular diffusion of critical regulatory ions and small molecules between contiguous cells and are normally present in large aggregates called “plaques” on the cell membrane [1,2]. Gap junction assembly is dependent on the efficient delivery of connexons (half of a gap junction) to the plasma membrane and subsequent docking of these between adjacent cells [3]. Gap junctions are then recycled from the centre of the plaques into the endosomal/lysosomal pathway, but they can also be degraded by the proteasome [4]. Regulation of gap junctional intercellular communication (GJIC) has been demonstrated to produce cellular changes underlying tumour formation. Additionally, connexons have been shown to have gap junction-independent tumour promoting activity [5].

There are 21 human connexin proteins, all of which have four transmembrane α helices anchored in the cell membrane with a short N- and variable length C-terminus in the cytoplasm [3]. For example, Connexin 43 (Cx43), the most widespread connexin and a major component of gap junctions in stratified epithelia, has a 151 amino acid long C-terminus which integrates with intracellular signalling pathways [6]. A body of evidence has accumulated to show that GJIC may be lost during malignant progression, as seen in HPV-positive cervical cancer [7]. Cx43 is often down-regulated in epithelial carcinomas [7] as well as precancerous lesions [8] although in other cases expression may be increased in invasive tumours [9]. Nevertheless, the steps leading to changes in connexin expression and trafficking and how these are related to tumour progression are largely unknown.

Human papillomaviruses (HPVs) are small double-stranded DNA viruses, which infect the stratified epithelia [10]. HPV16 is the most prevalent so-called “high-risk” HPV genotype associated with cervical and other anogenital carcinomas [11], in addition to a subset of head and neck cancers [12]. Progression from the premalignant to malignant phase of high-risk HPV-associated disease is driven by overexpression of the viral oncoproteins E6 and E7 [10]. In the nucleus, E6 binds and targets the tumour suppressor p53 for degradation [13]. However, E6 also contains a highly conserved C-terminal motif [14,15] that can interact with the PDZ (PSD-95/Dlg/ZO-1) domain-containing proteins MAGI-1, 2, 3, MUPP-1, hScrib and hDlg [16,17]. *In vitro* and *in vivo* studies have revealed that the E6 PDZ binding motif is essential for the HPV infectious life cycle and for HPV-associated tumour progression underlining the importance of E6/PDZ protein interactions [15,18].

Proteins of the membrane-associated guanylate kinase homologue (MAGUK) family can form protein scaffolds and comprise macromolecular complexes with protein partners thought to be involved in cell signalling cascades and cell morphology organization [19,20]. hDlg is a MAGUK protein located at intercellular contact sites in epithelial cells [21,22]. Previously we reported an interaction between Cx43 and hDlg in HPV16-positive cervical epithelial cells. The C-terminal domain of Cx43 binds the N- and C-termini of hDlg [23]. hDlg and Cx43 were both located at the plasma membrane in non-tumour cervical epithelial cells (W12G) but were co-localised in the cytoplasm in invasive cervical tumour cells derived from these (W12T; formerly named W12GPXY) [23,24]. Functional studies indicated that hDlg was responsible for maintaining a cytoplasmic pool of Cx43, protected from degradation that may be capable of trafficking to the membrane.

In this study we first demonstrate a physical association between hDlg and Cx43 *in vivo*. The known interaction of E6 with hDlg prompted us to investigate whether HPV16 E6 could interact with and regulate the subcellular location of the hDlg/Cx43 complex. We detected association of E6 with Cx43 at low levels in W12T cells. However, E6 siRNA depletion in W12T cells caused redistribution of Cx43 from the cytoplasm to the membrane. Conversely, overexpression of HPV 16 or 18 E6 in HPV-negative C33a cells resulted in reduced levels of Cx43 and redistribution from the membrane to the cytoplasm. Finally, HPV-negative C33a cells overexpressing a wild type or mutant HPV18 E6 demonstrated the critical role E6 plays in membrane localisation of Cx43. These findings reveal a novel E6-regulated pathway through which Cx43 trafficking, and potentially GJIC, is altered in cervical tumour cells.

## 2. Materials and Methods

### 2.1. Cell Culture and Antibodies

W12G cells are non-tumour cervical epithelial cells (clone 20861: [25]). W12T cells are invasive tumour cells derived from W12G cells. They were originally called W12GPXY [24]. W12T, C33a, and HEK-293 cells were cultured in Dulbecco’s modified Eagle’s medium (DMEM) supplemented with 10% FCS; W12E, W12G cells were cultured in serum-free keratinocyte growth medium (KGM) from Cambrex, UK (cc-3101). All cells were maintained under humidified 5% CO_2_ at 37 °C.

Polyclonal antibody raised in rabbits against a synthetic peptide corresponding to residues 363–382 of native Cx43, was kindly provided by Dr E. Rivedal, The Norwegian Radium Hospital, Oslo. Polyclonal antibodies against HPV-16 E6 (sc-1583) and against ZO-1 (61-7300) and a monoclonal antibody against hDlg (sc-9961) were purchased from Santa Cruz Biotechnology, California, CA, USA. Polyclonal antibody against flag (F7425) was purchased from Sigma (Poole, UK). Monoclonal antibody against Cx43 (C-6219) was purchased from Sigma. Antibody dilutions were as described [23].

### 2.2. Proximity Ligation Assay (PLA)

Archival paraffin-embedded cervical biopsy samples were obtained with ethical permission (Glasgow Royal Infirmary: RN04PC003). Diagnosis was made by two gynaehistopatholgists. HPV presence was confirmed by PCR. Sections on slides were de-paraffined, and antigen retrieval performed using sodium citrate (10 mM, pH6.0). Sections were incubated in blocking solution (PBS, 0.5% BSA, 0.25% TritonX-100) for 1 h and primary antibodies for Cx43 (Rivedal, 1:50) and hDlg (Santa Cruz, 1:50) added in blocking solution overnight at 4 °C. For standard immunofluorescence, samples were washed in PBS three times, blocked for 30 min, then secondary antibodies (Alexa Fluor, Lifetechnologies, Paisley, UK, 1:500) added for 30 min at room temperature. For Proximity Ligation Assay (PLA), following overnight primary antibody incubation, samples were washed in PBS and detection performed using a Duolink kit (Sigma, Poole, UK) as previously described [26]. All samples were mounted using ProLong antifade diamond containing DAPI (Life Techologies, Paisley, UK). Negative controls (no primary antibody) were included in all experiments. Images were taken using a Zeiss LSM510 Meta confocal microscope.

### 2.3. Co-Immunoprecipitation

Cells at 70% confluence were washed twice with ice-cold PBS and scraped into 5 mL chilled IP buffer containing PBS, 1% Triton X-100, 0.5% CHAPS, 0.1% SDS with one tablet of mini-proteinase inhibitor cocktail and one tablet of PhosSTOP phosphatase inhibitor cocktail per 10 mL (Roche, UK). The cell lysates were incubated on ice for 15 min and the solutions sonicated for 1 min on ice. Cell lysates were then cleared of cellular debris by centrifugation at 12,000 g for 10 min at 4 °C and the protein concentration determined by Bradford’s assay (Bio-Rad Laboratories, Hemel Hempstead, UK). Cell lysates were pre-cleared with protein-G sepharose beads (Sigma, Poole, UK) for one hour at 4 °C. Primary antibodies were then added to 100 µg protein of each cell lysate and incubated for 2 h at 4 °C with rotation. Subsequently, 40 µg of reconstituted protein G-Sepharose was added and the volume was adjusted to 750 µL.The samples were mixed by rotation at 4 °C for 4–5 h. Immunocomplexes were then harvested by centrifugation and washed four times with 250 µL ice-cold IP buffer and once with 25 µL ice cold PBS. Proteins were solubilised by sonicating the complexes in PBS mixed with 5 µL 6× protein loading buffer (1× buffer: 125 mM Tris (pH 6.8), 4% SDS, 20% glycerol, 10% mercaptoethanol and 0.006% bromophenol blue) for 5 min on ice and separated by SDS-PAGE.

### 2.4. Western Blotting

Confluent cells were scraped into protein-loading buffer (125 mM Tris (pH 6.8), 4% SDS, 20% glycerol, 10% mercaptoethanol and 0.006% bromophenol blue, fresh protein inhibitor cocktail (Roche, Welwyn Garden City, UK); 50 µg protein was resolved by polyacrylamide gel electrophoresis and subsequently transferred to a nitrocellulose membrane. The membrane was preincubated for 1 h at room temperature with 5% dried milk in PBS-0.1% Tween, before overnight incubation at 4 °C with diluted primary antibody in PBS-Tween, 1% dried milk. Horseradish peroxidase (HRP)-conjugated secondary antibodies were diluted 1:1000 in PBS-Tween and incubated for 1 h. The blot was developed using Pierce enhanced chemi-luminescence (ECL) kit and exposed to Kodak X-OMAT film.

### 2.5. Plasmid Construction and Cell Transfection

The HPV18 E6 wild type gene and E6 aa156 Thr → Glu mutation cloned into pcDNA-3 plasmid were provided by Dr Lawrence Banks [27]. The plasmids were sequentially digested with EcoR I and Hind III, then the E6 fragments were purified with phenol:chloroform extraction and ethanol precipitation. The vector p3x-FLAG-CMV^TM^-10 (Sigma, Poole, UK) was cut with EcoR I and Hind III and ligated with the wild type and mutant HPV18 E6 DNA fragments to generate the pN-terminus 3XFLAG-fused wild-type HPV18 E6 (pNFWE6) and pN-terminus 3X FLAG-fused mutant HPV 18 E6 (pNFME6). The recombined plasmids were sequenced to confirm correct insertion.

C33a cells (2 × 10^5^) were transfected with 3 µg of p3x-FLAG-CMV^TM^-10, pNFWE6 or pNFME6 using Lipofectamine (Invitrogen, Paisley, UK) according to the recommended protocol and selected in medium supplemented with 500 µg/mL G418 (Sigma, Poole, UK) to obtain stably transfected cell colonies. The experiments were carried out with 2 independent cell lines selected from each transfection. The plasmid pMAXGFP was used as an expression control plasmid and for calculating transfection efficiency.

### 2.6. W12T Cell Transfected with siRNA

Purified siRNA against HPV16 E6 (5′GUUACCACAGUUAUGCACATT3′) or a control siRNA (siGLO), were transfected into 1.5 × 10^5^ W12T cells per well in DMEM with 10% FCS using Lipofectamine RNAiMAX (Invitrogen, Paisley, UK) according to the manufacturer’s instructions. The final siRNA final concentration was 0.2 μM. Cells were harvested for analysis at 32 h post-transfection.

### 2.7. Immunofluorescence Microscopy

Cell were grown on sterile 18 × 18 mm coverslips until 90% confluent, washed three times with PBS and fixed and permeabilised with 100% ice-cold methanol for 5 min at −20 °C or with 58 mM sucrose, 4% formaldehyde in PBS for 10 min at room temperature [23]. Coverslips were blocked using 5% (*v*/*v*) horse serum in PBS for 30 min at room temperature then washed three times with PBS. Cells were incubated with diluted primary antibodies in 1% horse serum in PBS for 1 h at room temperature. Coverslips were washed in PBS six times before incubation for 1 h with secondary antibodies labelled with fluorescein or Texas-Red diluted 1:100 in PBS (Vector laboratories, Peterborough, UK). After washing in PBS six times, the coverslips were mounted with Vectashield mounting medium (with DAPI as a nuclear stain). Negative controls (no primary antibody) were included in all experiments. Images were taken using a Zeiss LSM510 Meta confocal microscope.

## 3. Results

### 3.1. hDlg and Cx43 Interact *In Vivo*

**Figure 1:**
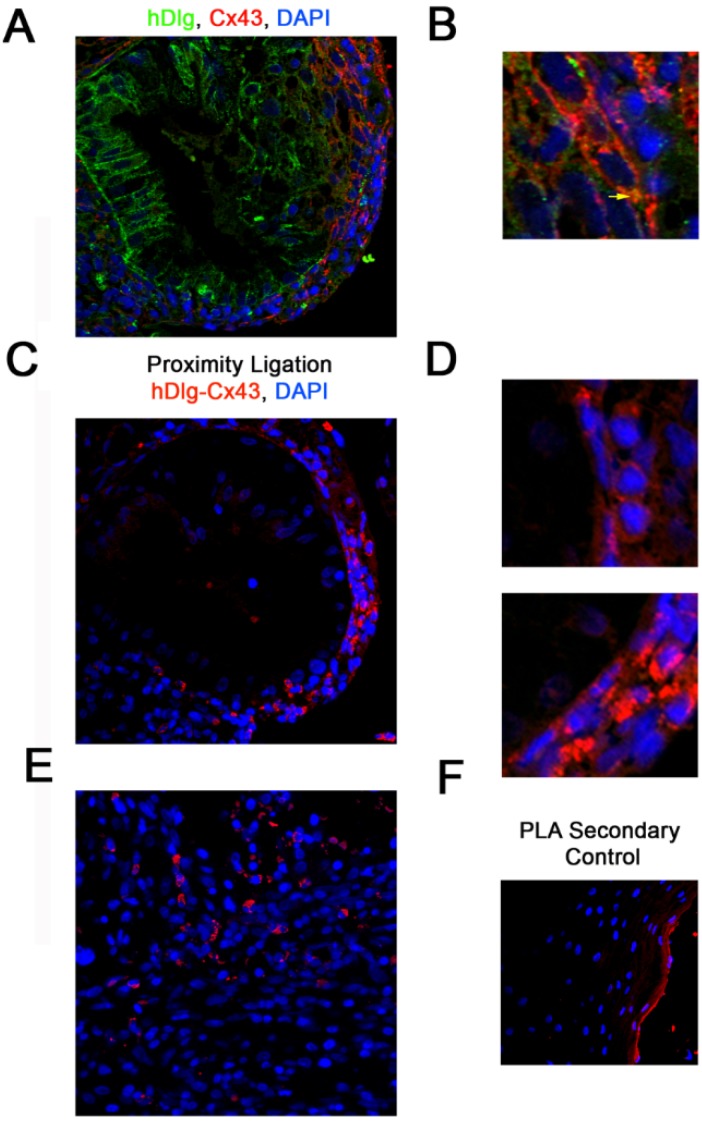
Cx43 and hDlg interact directly in cervical tumour cells *in vivo*. (**A**) Representative immunofluorescence image showing a section of a high grade cervical lesion; (**B**) A region of Cx43 and hDlg colocalisation in (**A**) enlarged 5×. Immunofluorescence shows hDlg (green), Cx43 (red) and DAPI (blue). The yellow arrow indicates one area of Cx43/hDlg colocalisation; (**C**) Immunofluorescence proximity ligation for hDlg and Cx43, where red staining indicates colocalisation of the proteins within 40 nm of each other; (**D**) Regions of Cx43 and hDlg colocalisation detected by PLA in C enlarged 5×; (**E**) Immunofluorescence proximity ligation for hDlg and Cx43 in a region of a cervical tumour; (**F**) Immunofluorescence proximity ligation secondary control on the outer region of the cervical lesion a part of which is shown in (**C**).

Previously we demonstrated loss of GJIC in a number of HPV16-positive (CaSki, SiHa, W12T (formerly named W12GPXY)) and HPV18-positive (HeLa) cervical cancer cell lines but not in HPV16-positive non-tumour cervical epithelial cells (W12E, W12G) [23,24]. In the W12T cervical tumour cells Cx43 was no longer located in membrane gap junction plaques but was redistributed to the cytoplasm where it colocalised with the PDZ domain protein hDlg. Cx43 was also located in the cytoplasm in two other HPV-16-positive cervical cancer cell lines (CaSki and SiHa cells (Supplementary Figure S2). To determine if the Cx43/hDlg interaction occurs in cervical epithelial tissues *in vivo* we examined location of the proteins in HPV16-positive high grade cervical lesions. Analysis by immunofluorescence showed that hDlg and Cx43 co-localise in epithelial cells in discrete regions of the cells *i.e.*, perinuclear location (Figure 1A,B). To further define this, we probed tissues using a proximity ligation assay (PLA), which identifies protein interactions based on proteins being within 40 nm of each other. Figure 1C,D (red spots) shows detection of the hDlg-Cx43 complex within the epithelial cells of the cervical lesion. Figure 1E shows low level detection of Cx43/hDlg complexes in a limited area of a representative cervical tumour in agreement with the observation that Cx43 levels decline in cervical cancer tissues *in vitro* and *in vivo* [24,28,29]. Two cervical lesions and two cervical cancers were examined and there was evidence that Cx43 and hDlg were in close proximity in all tissues. Figure 1F shows a duolink secondary control. The image is from the outer region of the tissue shown in Figure 1C. We chose this area of the tissue because it represents the only autofluorescence we detected in any of the tissues we examined. Some antibody trapping on the outer surface of the epithelium was detected but there was no staining detected in the cells in the tissue interior. These data confirm our previous *in vitro* findings that Cx43 and hDlg interact and demonstrates the formation of protein complexes in human cervical epithelial cells *in vivo*.

### 3.2. HPV16 E6 Is Present in a Complex Containing hDlg and Cx43 Cervical Tumour Cells

**Figure 2:**
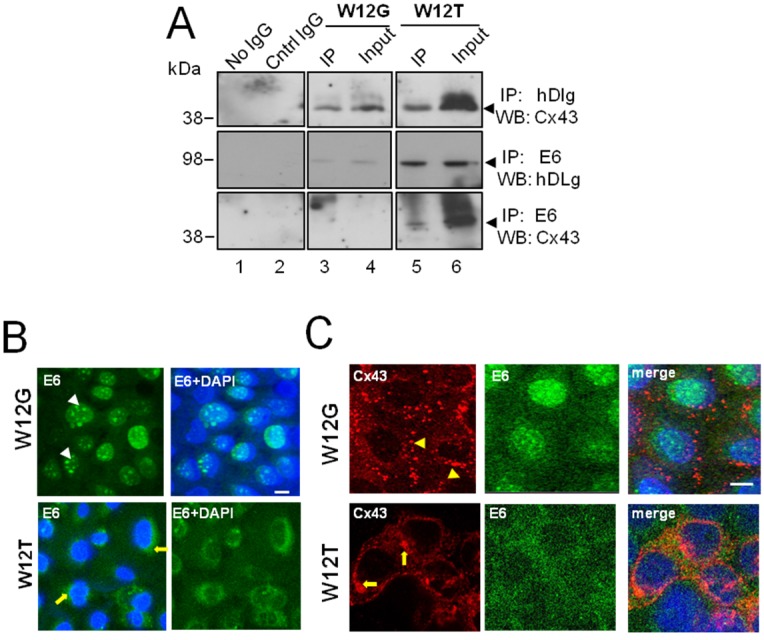
HPV16 E6 associates with Cx43 in W12T cervical cancer cells. (**A**) Co-immunoprecipitation of E6 with hDlg and Cx43 in W12G non-tumour and W12T tumour cells. Antibodies used in immunoprecipitation (IP) and the antibodies used to probe western blots (WB) are indicated on the right hand side. No IgG, beads alone used in the immunoprecipitation. Cntr IgG, a matched antibody isotype negative control immunoprecipitation. Input, 10% of the volume of cellular extract as used in the co-immunoprecipitation experiments; (**B**) Confocal immunofluorescence microscopy imaging showing E6 (green) in the nucleus of W12G cells (arrowheads) but in the cytoplasm of W12T cells (arrows); (**C**) Confocal immunofluorescence microscopy imaging of Cx43 (red) in W12G cells located in large punctuate gap junction plaques (arrowheads) on the membrane. In W12T cells membrane Cx43 (red) is reduced and some perinuclear staining is detected (arrows). E6 is shown in green. Nuclei are stained with DAPI (blue). Bar = 10 µM.

Structural analysis has demonstrated that PDZ domains in hDlg can bind to the X-S/T-X-V/L protein motif of high risk HPV E6 proteins [14]. As we have shown previously that the C-terminal domain of Cx43 interacts with the N- and C- termini of hDlg [23], it is possible that E6, a 17 kDa protein, could form a complex with hDlg while hDlg is also bound to Cx43. Therefore we investigated formation of protein complexes between Cx43, hDlg and E6 by co-immunoprecipitation in W12G (non-tumour cervical epithelial cells) and in W12T (cervical tumour) cells (Figure 2).

Cx43 and hDlg were co-immunoprecipitated from both W12G (top panel, lane 3) and W12T (top panel, lane 5) cell extracts (Figure 2A). Moreover, as expected, E6/hDlg complexes were able to be precipitated from both W12G and W12T cells (Figure 2A middle panel, lanes 3 and 5) but were more abundant in the latter. Co-immunoprecipitation of Cx43 with E6 antibody was detected at low levels in W12T cells (Figure 2A, bottom panel, lane 5) but not in W12G cells. A reverse precipitation of E6 with hDlg or Cx43 was not successful due to poor reactivity of the HPV16 E6 antibody in western blots. These data indicate that in cervical tumour cells E6 can be part of a complex containing hDlg and Cx43, at least at low levels.

Next we used confocal immunofluorescence microscopy to investigate the subcellular location of endogenous Cx43 and E6 in the W12 non-tumour and tumour cells. In the W12G non-tumour cells E6 was primarily localised within the nucleus as expected (Figure 2B, arrowheads) [30]. However, in the W12T tumour cells E6 was located in both the nucleus and the cytoplasm (Figure 2B, arrows). Immunofluorescence analysis of E6 and Cx43 in the two cell types revealed that Cx43 was located mainly in gap junction plaques (arrowheads) at the plasma membrane in W12G cells. There was no colocalisation of E6 and Cx43 as they were detected in different subcellular compartments (Figure 2C). However, in W12T cells Cx43 was located in a perinuclear location in the cytoplasm as previously observed (arrows) [23,24] and E6 was diffusely located in the cytoplasm (Figure 2C). E6 and Cx43 colocalised around the nucleus of W12T cells

### 3.3. HPV16 E6 Controls Cx43 Trafficking in Cervical Tumour Cells

**Figure 3:**
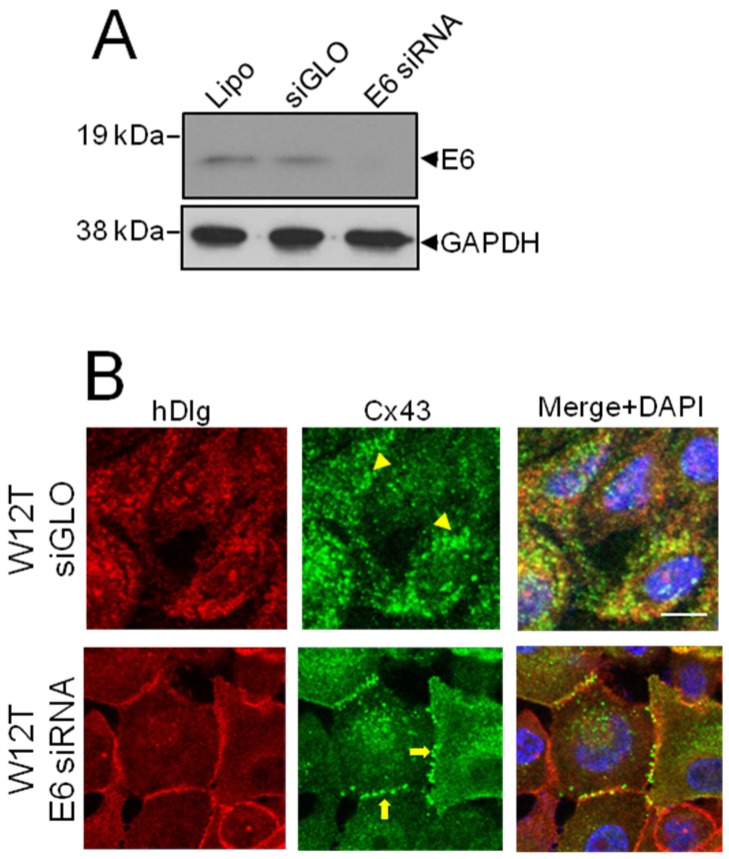
E6 restricts Cx43 trafficking to the plasma membrane in W12T cells. (**A**) Western blot showing depletion of E6 following siRNA treatment of W12T cells. Lipo, mock transfected cells treated with lipofectamine. siGLO, cells transfected with control siRNA. E6 siRNA, cells transfected with HPV16 E6 siRNA; (**B**) Confocal immunofluorescence microscopy imaging showing the location of hDlg (red) and Cx43 (green) in control transfected W12T cells or in W12T cells transfected with siRNA against E6. Arrowheads indicate cytoplasmic Cx43 while arrows indicate Cx43 located in gap junctions plaques on the cell membrane. Nuclei are stained with DAPI. Bar = 10 µM.

Since our previous study indicated that hDlg was not responsible for Cx43 relocation from the membrane to the cytoplasm [23] next, we investigated the hypothesis that if E6 can potentially form a complex with hDlg and Cx43 it could restrict Cx43 trafficking to the plasma membrane. W12G and W12T are isogenic cell lines where the HPV16 genome is integrated into the host genome. Both lines express only the bicistronic E6E7 viral mRNA [25,31]. Therefore, we depleted the E6E7 mRNA using siRNA and examined changes in the subcellular location of Cx43 in W12T cells. Western blotting showed that specific siRNA treatment caused a marked reduction in total E6 protein levels (Figure 3A). Confocal immunofluorescence microscopy showed that treatment of W12T cells with a control siRNA (siGLO) did not alter the cytoplasmic location of Cx43 (Figure 3B arrowheads). In contrast, treatment of W12T cells with the E6 siRNA resulted in some restoration of Cx43 on the plasma membrane in typical gap junction plaques at points of cell-cell contact (Figure 3C, arrows). hDlg also appeared at the membrane in the E6 siRNA-treated cells (Figure 3C). These data indicate that a protein translated from the E6E7 bicistronic mRNA may be involved in Cx43 (and hDlg) intracellular trafficking. There are a number of possible proteins: E6 full length, E6*I, E6*II, E6*X and E7 [32]. However, only E6 full length can bind hDlg. Next we tested whether this protein was responsible for controlling Cx43.

### 3.4. HPV E6 Causes Reduced Levels of Cx43 in C33a Cells

**Figure 4:**
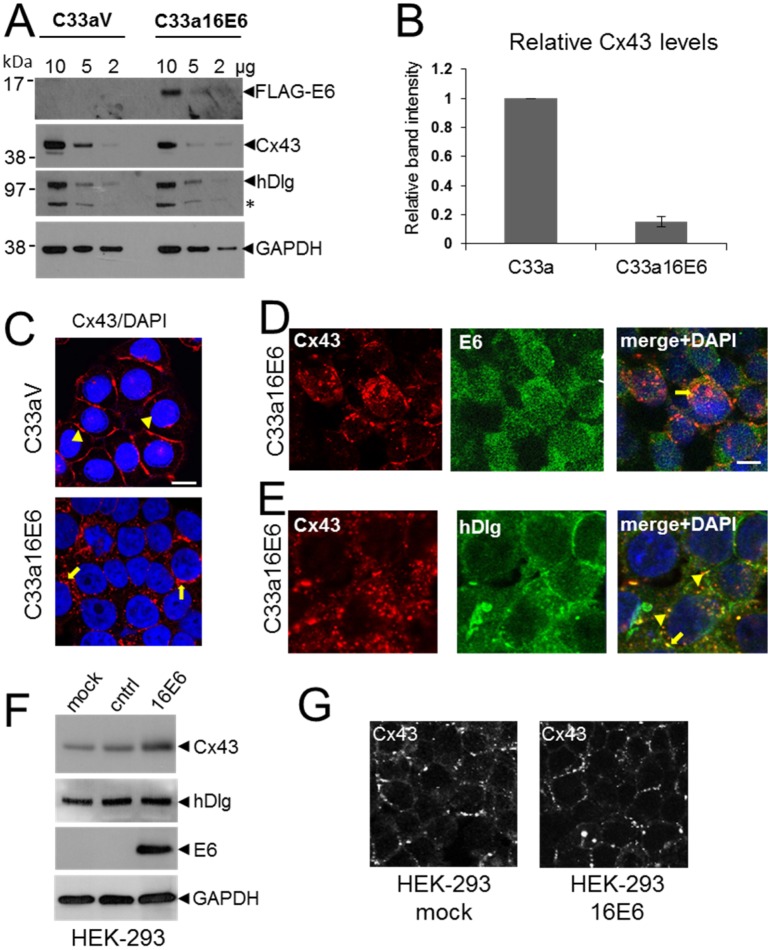
C33a cells expressing HPV16E6 show reduced Cx43 levels and some colocalisation between E6 and Cx43 is seen in the cytoplasm. (**A**) Titration western blot showing the levels of Cx43 and hDlg in C33a cells stably transfected with vector alone (C33aV) or with a vector expressing FLAG-tagged HPV16 E6 (C33a16E6). As indicated, 10, 5 or 2 µg protein extract was applied to the lanes as indicated. hDlg degradation results in a product that appears as an additional protein species of around 70 kDa (asterisk). E6 was detected in C33a16E6 cells using a FLAG antibody. GAPDH is shown as a loading control; (**B**) Quantification of levels of Cx43 relative to GAPDH from 5 separate western blot experiments. The data show the mean and standard error of the mean; (**C**) Confocal immunofluorescence microscopy imaging of Cx43 (red) location in C33aV and C33a16E6 cells; (**D**) Confocal immunofluorescence microscopy imaging of the location of Cx43 (red) and E6 (green) in C33a16E6 cells. Cytoplasmic colocalisation is indicated (arrow). The arrowhead indicates some Cx43 remaining on the plasma membrane where there is also co-staining with E6; (**E**) Location of Cx43 (red) and hDlg (green) in C33a16E6 cells. Co-staining in the cytoplasm is indicated with arrowheads. Nuclei are stained with DAPI. Bar = 10 µM; (**F**) Western blot analysis of Cx43 and hDlg levels in HEK-293 cells expressing HPV16 E6. Mock, mock transfected cells, cntrl, cells transfected with a control plasmid, 16E6, cells transfected with an expression construct for HPV16 E6. (**G**) Confocal microscopy analysis of Cx43 location in 293 cells transfected with the control plasmid (mock) or with an expression construct for HPV16 E6 (16E6).

Previously we found that cytoplasmic hDlg protected a pool of Cx43 from degradation: siRNA depletion of hDlg resulted in reduced levels of Cx43 in W12T cells [23]. Although not as efficient as HPV18 E6, HPV16 E6 can target hDlg for degradation via the proteasome in cervical cancer cells [27]. Thus it is possible that the reduction in Cx43 levels following depletion of hDlg could be mediated by HPV E6. To test this we examined Cx43 expression in HPV-negative C33a cervical tumour cells stably transfected with either an empty FLAG vector (C33aV, no E6 expression) or a vector expressing FLAG-tagged HPV16 E6 (C33a16E6). Levels of Cx43 protein were assessed using a protein lysate titration western blot and quantified (Figure 4A,B). As expected FLAG-E6 was detected using an anti-FLAG antibody in C33a16E6 cells but not in C33aV cells (Figure 4A). Expression of E6 in C33a16E6 cells resulted in a significant reduction in Cx43 expression (Figure 4A,B) with no notable changes in the expression of hDlg (Figure 4A). These data suggest that E6 may target Cx43 for degradation independent of its effects on hDlg.

C33a cells normally retain strong GJIC and display Cx43 gap junction plaques on the plasma membrane [24] indicating that HPV expression might regulate GJIC in tumour cells. To confirm our data that E6 regulates Cx43 intracellular trafficking we examined the effect of E6 expression on Cx43 and hDlg in C33a cells using confocal immunofluorescence microscopy. Figure 4C (arrowheads) shows that Cx43 was located primarily on the plasma membranes between adjacent C33aV cells. In contrast, in C33a16E6 cells Cx43 was primarily localised within intracellular regions (Figure 4C arrows), with a marked reduction in membrane gap junction plaques. Figure 4D show that in these cells E6 was located throughout the cytoplasm and the nucleus with some evidence of colocalisation with Cx43 on the plasma membrane (arrowhead) and in the cytoplasm (arrow). In contrast to what we observed in W12T cells, hDlg was found mainly at the cell periphery and there was only limited colocalisation of hDlg with Cx43 in the cytoplasm of C33a16E6 cells (Figure 4E). To test the effect of ectopic expression of HPV16 E6 on Cx43 levels in a non-cervical tumour cell line we transfected the FLAGE6 expression vector into HEK-293 cells. Figure 4F shows that no change in hDlg levels was detected when E6 was expressed and instead of a decrease in Cx43 levels a slight increase was observed. E6 expression did not cause Cx43 relocation from the membrane in these cells (Figure 4G).

### 3.5. E6 PDZ Binding Motif Is Required for Relocation of Cx43 from the Membrane to the Cytoplasm

**Figure 5:**
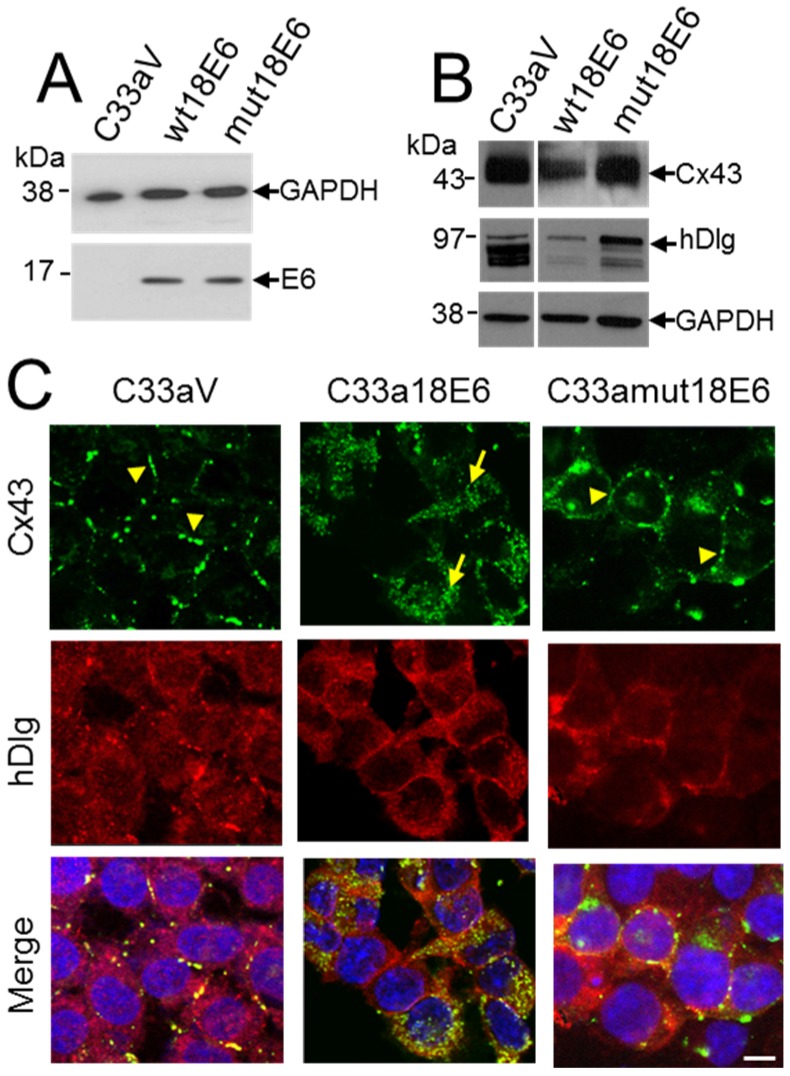
C33a cells expressing a mutant HPV18 E6 that cannot bind hDlg display membrane Cx43. (**A**) Western blot analysis of FLAG E6 expression in C33a cells stably transfected with vector alone (C33aV), vector expressing HPV18E6 (wt18E6) or a mutant HPV18 E6 that does not bind hDlg (mut18E6); (**B**) Western blot analysis of levels of Cx43 and hDlg in C33aV, C33a wt18E6 and C33a mut18E6. GAPDH is used as a loading control for the blots in (**A**,**B**); (**C**) Confocal immunofluorescence microscopy imaging of Cx43 (green) and hDlg (red) in C33aV cells, C33a18E6 cells and C33amut18E6 cells (cells expressing C-terminal mutated 18E6 that cannot bind hDlg). Membrane gap junction plaques are indicated with arrowheads. The arrow in B indicates cytoplasmic Cx43 staining. Nuclei are stained with DAPI. Bar = 10 µM.

To investigate the relationship between E6 and hDlg and Cx43 re-location to the cytoplasm in cervical tumour cells, C33a cells were transfected with empty vector (C33aV) or a vector expressing wild type E6 (C33a18E6) or the same vector expressing an HPV18 E6 mutant that does not bind hDlg [27] (Figure 5A). This time HPV18 E6 was used because it binds and degrades hDlg more effectively than HPV16 E6 [33]. Moreover, there is a high degree of conservation between the HPV18 and HPV16 E6 proteins and loss of Cx43 GJIC is observed in HPV18 as well as HPV16-positive cervical tumour cells [24]. HPV18 E6 expression caused a reduction in both Cx43 and hDlg levels but expression of the HPV18 E6 mutant that does not bind hDlg did not affect levels of either protein compared to cells transfected with vector alone (Figure 5B). Similar to what was observed for HPV16 E6, Cx43 relocated from the cell membrane of C33aV cells (Figure 5C) to a perinuclear location in the C33a cells expressing ectopic 18E6 (Figure 5C). hDlg was present at the membrane and in the cytoplasm of both the C33aV and C33a18E6 cells but displayed increased colocalisation with Cx43 in the C33a cells expressing 18E6 (Figure 5C).

Our data suggest that E6 is involved in Cx43 trafficking and turnover in cervical cancer cells. E6 could control Cx43 via changes in intracellular signalling that impact on the Cx43 cytoplasmic C-terminal domain. Alternatively, E6 could regulate Cx43 via its effects on hDlg. If hDlg-E6 interaction was important in Cx43 recycling from the membrane then a mutant E6 that could no longer bind hDlg might alter Cx43 trafficking. To test this, we examined the effects on Cx43 localisation in C33a cells of the HPV18 E6 mutated in its hDlg binding motif (C33amut18E6) [27] (Figure 5C). In contrast to wild type E6 transfected cells (C33a18E6 cells) where Cx43 and hDlg were located in the cytoplasm and Cx43 was found in a perinuclear location (Figure 5C), C33a cells expressing the mutant E6 displayed some Cx43 on the cell membrane in gap junction plaques. Taken together these data reveal that high risk HPV E6 controls critical steps in Cx43 recycling from the plasma membrane and, at least for HPV18, that E6 interaction with hDlg may underlie the loss of GJIC observed in cervical tumour cells.

## 4. Discussion

Elucidation of the control of Cx43 trafficking is crucial in understanding gap junction and connexin hemichannel assembly and disassembly, and its impact in a range of skin diseases, cardiovascular disease, diabetes and cancer. Although both reduced levels and overexpression of Cx43 have been reported in various cancers [7], loss of Cx43 from the plasma membrane has been observed in cervical precancers and cancers [8]. These studies suggest that Cx43 trafficking is altered in cervical cancer cells.

Previous studies on Cx43 trafficking have focused on the interaction of Cx43 with the membrane-associated scaffolding protein ZO-1. ZO-1 was shown to control cell migration, adhesion and Cx43 trafficking to and from the membrane in a range of cell types [34,35,36,37,38,39]. However, we discovered that Cx43 interacts with another scaffolding protein hDlg, in HPV16-positive cervical tumour cells. We have now confirmed this interaction can occur *in vivo* suggesting it has a functional significance. hDlg appears to have a role in Cx43 trafficking by maintaining a cytoplasmic pool of Cx43 protected from lysosomal degradation [23].

GJIC is disrupted and Cx43 is located in the cytoplasm of HPV-positive cervical tumour cells but not in HPV-negative cervical tumour cells [24]. This indicates that one of the viral oncoproteins could control Cx43 trafficking. High risk HPV E6 oncoprotein is the most obvious candidate because it associates with hDlg through its C-terminal PDZ binding motif [14,15], and we have evidence that Cx43 forms a complex with hDlg [23]. Because the amounts and intracellular locations of HPV16 E6 were significantly different between W12G non-tumour and the W12T tumour cells we considered the hypothesis that cytoplasmic E6 in W12T cells could modulate Cx43 and/or hDlg trafficking to the plasma membrane. Our data suggest that E6 does not appear to have a very strong interaction with Cx43 because it was detected only at low levels in co-immunoprecipitation in cervical tumour cells. This result is perhaps not surprising given that HPV16 E6 has been shown to interact with hDlg only at low affinity [40]. Some co-localisation of E6 with Cx43 was detected in the cytoplasm of W12T and in C33a cells ectopically expressing FLAG-tagged E6, but because E6 was distributed widely in the cytoplasm the specificity of this co-localisation requires further investigation. The data suggest that Cx43, hDlg and E6 interact in HPV-positive cervical tumour cells. It seems likely that Cx43 interaction with E6 is mediated by E6 binding hDlg.

siRNA depletion of E6 in W12T cells led to relocation of some Cx43 onto the plasma membrane where it formed gap junction plaques. This suggests that E6 is involved in the trafficking of Cx43. E6 can control intracellular signalling pathways [41,42,43] that impact upon Cx43 trafficking [6]. Therefore, increased E6 levels could affect Cx43 trafficking indirectly via changes in intracellular signalling sensed by the C-terminal intracellular domain of Cx43. Such changes may include post-translation modifications such as phosphorylation. Indeed dephosphorylation of Cx43 is known to lead to loss of Cx43 from gap junction plaques and intracellular relocation [44,45,46] and phosphorylation levels of Cx43 are lower in W12T cells than in W12G cells (Supplementary Figure S1). Alternatively, formation of an intracellular Cx43/hDlg/E6 complex in the E6-expressing cervical tumour cells might inhibit Cx43 trafficking to the membrane directly by removing Cx43 from its normal trafficking pathway. In our experiments HPV18 E6/hDlg interaction seemed to be important for Cx43 trafficking because it was found on the plasma membrane in HPV-negative C33a cells expressing the hDlg binding mutant of HPV18 E6 Cx43. It is also possible that hDlg could function in the transport cycle of Cx43 by providing a docking platform for molecules [47] in addition to E6 that are involved in assembling or disassembling connexon hemichannels. Expression or activity of such molecules could be induced by increased expression of E6 and/or by other changes induced by tumour progression.

It has been suggested that changes in Cx43 phosphorylation can be induced by changes in tissue architecture [48]. Compared to W12G cells, W12T cells exhibit alterations in microtubules and microfilaments and drug-induced disruption of the cytoskeleton led to changes in Cx43 cellular location (unpublished data). Interestingly, no relocation of Cx43 was observed in HEK-293 cells expressing HPV16 E6. It is possible that Cx43 post-translation modifications in HEK-293 cells result in a protein that is not able to dock with hDlg (or other trafficking proteins) or that signalling pathways in HEK-293 cells cannot be altered by E6 in a manner similar to those in W12T or C33a cells. It will be interesting to examine Cx43 and hDlg localization in the normal cervix and the HPV-infected cervix. For example, the transformation zone that has altered architecture and appears to be susceptible to HPV infection may exhibit Cx43/hDlg colocalisation. The relative contribution of E6/hDlg interaction, Cx43 phosphorylation and cytoskeletal alterations to Cx43 trafficking remains to be tested in normal and tumour tissues.

Ectopic expression of E6 in C33a cervical tumour cells resulted in reduced levels of Cx43. These data suggest that E6 is involved in targeting Cx43 for degradation. This observation correlates with the fact that in W12T cells that express higher levels of E6, but not in the W12G cells that express lower levels, Cx43 entered, and was degraded by, the lysosomal degradation pathway [23]. A study using clones of HPV-18 positive HeLa cervical tumour cells revealed a spectrum of Cx43 expression with the majority displaying no or low Cx43 levels in agreement with our data [28]. Similarly, in C33a cells expressing HPV18 E6 Cx43 was located in the cytoplasm and few gap junction plaques were observed. In contrast, King *et al.* showed that overexpression of Cx43 in HeLa cells resulted in appearance of gap junction plaques on the membrane [29]. However, Cx43 was also strongly expressed in the nucleus and cytoplasm, presumably due to the very high levels of Cx43 overexpression [29]. In this case endogenous HPV18 E6 could not mediate full cytoplasmic internalization of Cx43 perhaps due to the large amount of Cx43 in the cell overwhelming trafficking pathways. In HEK-293 cells overexpression of HPV16 E6 did not cause a reduction in Cx43 levels. This suggests that the effect of E6 is cell type-specific and perhaps restricted to cancer cells, for example Cx43 is also not degraded in W12G cells [23]. We have not yet tested whether Cx43 is targeted for lysosomal degradation in C33aE6 cells. Critically, Cx43 can also be degraded by the proteasome. E6 targets p53 for proteasomal degradation via the ubiquitin ligase, E6-associated protein (E6AP). It is possible that via E6, E6AP may also be able to ubiquitinate Cx43 and target it for degradation.

## 5. Conclusions

In conclusion, our findings highlight a role for high risk HPV E6 in Cx43 trafficking between the plasma membrane and the cytoplasm. Taken together, the present results suggest that when expressed at high levels, for example in cervical cancer cells, high risk HPV E6 may regulate Cx43 trafficking, resulting in decreased delivery of connexon hemichannels to the plasma membrane and inhibition of the formation of gap junctions. Alternatively, E6 could control rapid recycling of gap junction Cx43 into the cytoplasm. Whichever route is correct, reduced gap junctional communication may be another pathway by which E6 overexpression leads to tumour progression.

## Supplementary Files

Supplementary File 1

## References

[1] Evans W.H., Martin P.E. (2002). Gap junctions: Structure and function. Mol. Membr. Biol..

[2] Saez J.C., Berthoud V.M., Brañes M.C., Martínez A.D., Beyer E.C. (2003). Plasma membrane channels formed by connexins: Their regulation and functions. Physiol. Rev..

[3] Laird D.W. (2006). Life cycle of connexins in health and disease. Biochem. J..

[4] Kjenseth A., Fykerud T., Rivedal E., Leithe E. (2010). Regulation of gap junction intercellular communication by the ubiquitin system. Cell. Signal..

[5] Evans W.H., de Vuyst E., Leybaert L. (2006). The gap junction cellular internet: Connexin hemichannels enter the signalling limelight. Biochem. J..

[6] Johnstone S.R., Billaud M., Lohman A.W., Taddeo E.P., Isakson B.E. (2012). Posttranslational modifications in connexins and pannexins. J. Membr. Biol..

[7] Aasen T. (2015). Connexins: Junctional and non-junctional modulators of proliferation. Cell Tissue Res..

[8] Aasen T., Graham S.V., Edward M., Hodgins M.B. (2005). Reduced expression of multiple gap junction proteins is a feature of cervical dysplasia. Mol. Cancer.

[9] Jamieson S., Going J.J., D’Arcy R., George W.D. (1998). Expression of gap junction proteins connexin 26 and connexin 43 in normal human breast and in breast tumours. J. Pathol..

[10] Bodily J., Laimins L.A. (2011). Persistence of human papillomavirus infection: Keys to malignant progression. Trends Microbiol..

[11] Zur Hausen H. (2009). Papillomaviruses in the causation of human cancers—A brief historical account. Virology.

[12] Goon P.K., Stanley M.A., Ebmeyer J., Steinstrasser L., Upile T., Jerjes W., Bernal-Sprekelsen M., Gorner M., Sudhoff H.H. (2009). HPV and head and neck cancer: A descriptive update. Head Neck Oncol..

[13] Scheffner M., Werness B.A., Huibregtse J.M., Levine A.J., Howley P.M. (1990). The E6 oncoprotein encoded by human papillomavirus types 16 and 18 promotes the degradation of p53. Cell.

[14] Kiyono T., Hiraiwa A., Fujita M., Hayashi Y., Akiyama T., Ishibashi M. (1997). Binding of high-risk human papillomavirus E6 oncoproteins to the human homologue of the *Drosophila* discs large tumor suppressor protein. Proc. Natl. Acad. Sci. USA.

[15] Lee S.S., Weiss R.S., Javier R.T. (1997). Binding of human virus oncoproteins to hDlg/SAP97, a mammalian homolog of the *Drosophila* discs large tumor suppressor protein. Proc. Natl. Acad. Sci. USA.

[16] Subbaiah V.K., Kranjec C., Thomas M., Banks L. (2011). PDZ domains: The building blocks regulating tumorigenesis. Biochem. J..

[17] Roberts S., Delury C., Marsh E. (2012). The PDZ protein discs-large (DLG): The “Jekyll and Hyde” of the epithelial polarity proteins. FEBS J..

[18] Nicolaides L., Davy C., Raj K., Kranjec C., Banks L., Doorbar J. (2011). Stabilization of HPV16 E6 protein by PDZ proteins, and potential implications for genome maintenance. Virology.

[19] Dimitratos S.D., Woods D.F., Stathakis D., Bryant P.J. (1999). Signalling pathways are focused at specialised regions of the plasma membrane by scaffolding proteins of the MAGUK family. Bioessays.

[20] Kim E., Sheng M. (2004). PDZ domain proteins of synapses. Nat. Rev. Neurosci..

[21] Woods D.F., Bryant P.J. (1991). The disc-large tumor supressor gene of drosophila encodes a guanylate kinase homolog localized at septate junctions. Cell.

[22] Bilder D., Perrimon N. (2000). Localisation of apical determinants by the basolateral PDZ protein Scribbled. Nature.

[23] MacDonald A.I., Sun P., Hernandez-Lopez H., Aasen T., Hodgins M.B., Edward M., Roberts S., Massimi P., Thomas M., Banks L. (2012). A functional interaction between the MAGUK protein hDlg and the gap junction protein connexin 43 in cervical tumour cells. Biochem. J..

[24] Aasen T., Hodgins M.B., Edward M., Graham S.V. (2003). The relationship between connexins, gap junctions, tissue architecture and tumour invasion, as studied in a novel *in vitro* model of HPV-16-associated cervical cancer progression. Oncogene.

[25] Jeon S., Allen-Hoffman B.L., Lambert P.F. (1995). Integration of human papillomavirus type 16 into the human genome correlates with a selective growth advantage of cells. J. Virol..

[26] Johnstone S.R., Kroncke B.M., Straub A.C., Best A.K., Dunn C.A., Mitchell L.A., Peskova Y., Nakamoto R.K., Koval M., Lo C.W. (2012). MAPK phosphorylation of connexin 43 promotes binding of cyclin E and smooth muscle cell proliferation. Circ. Res..

[27] Gardiol D., Kühne C., Glausinger B., Lee S., Javier R., Banks L. (1999). Oncogenic human papillomavirus E6 proteins target the discs large tumour suppressor for proteasome-mediated degradation. Oncogene.

[28] King T.J., Fukushima L.H., Donlon T.A., Hieber A.D., Shimabukuro K.A., Bertram J.S. (2000). Correlation between growth control, neoplastic potential and endogenous connexin43 expression in HeLa cell lines: Implications for tumour progression. Carcinogenesis.

[29] King T.J., Fukushima L.H., Hieber A.D., Shimabukuro K.A., Sakr W.A., Bertram J.S. (2000). Reduced levels of connexin 43 in cervical dysplasia: Inducible expression in a cervical carcinoma line decreases neoplastic potential with implications for tumour progression. Carcinogenesis.

[30] Jackson R., Togtema M., Zehbe I. (2013). Subcellular localization and quantitation of the human papillomavirus type 16 E6 oncoprotein through immunocytochemistry detection. Virology.

[31] Jeon S., Lambert P.F. (1995). Integration of human papillomavirus type 16 DNA into the human genome leads to increased stability of E6 and E7 mRNAs: Implications for cervical carcinogenesis. Proc. Natl. Acad. Sci. USA.

[32] McFarlane M., MacDonald A.I., Stevenson A., Graham S.V. (2015). Human Papillomavirus 16 Oncoprotein expression is controlled by the cellular splicing factor SRSF2 (SC35). J. Virol..

[33] Thomas M., Glaunsinger B., Pim D., Javier R., Banks L. (2001). HPV E6 and MAGUK protein interactions: Determination of the molecular basis for specific protein recognition and degradation. Oncogene.

[34] Giepmans B.N.G., Moolenar W.H. (1998). The gap junction protein connexin43 interacts with the second PDZ domain of the zona occulens-1 protein. Curr. Biol..

[35] Toyofuku T., Yabuki M., Otsu K., Kuzuya T., Hori M., Tada M.H. (1998). Direct association of the gap junction protein connexin-43 with ZO-1 in cardiac myocytes. J. Biol. Chem..

[36] Hunter A.W., Barker R., Zhu C., Gourdie R. (2005). Zonal Occulens-1 alters connexin43 gap junction size and organisation by influencing channel accretion. Mol. Biol. Cell.

[37] Barker R.J., Price R.L., Gourdie R.G. (2002). Increased association of ZO-1 with connexin43 during remodeling of cardiac gap junctions. Circ. Res..

[38] Segretain D., Decrouy X., Dompierre J., Escalier D., Rahman N., Fiorini C., Mograbi B., Siffroi J.-P., Huhtaniemi I., Fenichel O. (2003). Sequestration of connexin43 in the early endosomes: An early event of Leydig cell tumor progression. Mol. Carcinogen..

[39] Laing J.G., Chou B.C., Steinberg T.H. (2005). ZO-1 alters the plasma membrane localization and function of Cx43 in osteoblastic cells. J. Cell. Sci..

[40] Thomas M., Massimi P., Navarro C., Borg J.-P., Banks L. (2005). The hScrib/Dlg apico-basal control complex is differentially targeted by HPV-16 and HPV-18 E6 proteins. Oncogene.

[41] Contreras-Paredes A., de la Cruz-Hernandez E., Martinez-Ramirez I., Duenas-Gonzalez A., Lizano M. (2009). E6 variants of human papillomavirus 18 differentially modulate the protein kinase B/phosphatidylinositol 3-kinase (akt/PI3K) signaling pathway. Virology.

[42] Spangle J.M., Munger K. (2010). The Human Papillomavirus Type 16 E6 Oncoprotein activates mTORC1 signaling and increases protein synthesis. J. Virol..

[43] Spangle J.M., Munger K. (2013). The HPV16 E6 oncoprotein causes prolonged receptor protein tyrosine kinase signaling and enhances internalization of phosphorylated receptor species. PLoS Pathog..

[44] Laird D.W., Castillo M., Kasprzak L. (1995). Gap junction turnover, intracellular trafficking, and phosphorylation of connexin43 in brefeldin A-treated rat mammary tumor cells. J. Cell Biol..

[45] Solan J.L., Lampe P.D. (2009). Connexin43 phosphorylation: Structural changes and biological effects. Biochem. J..

[46] Beardslee M.A., Laing J.G., Beyer E.C., Saffitz J.E. (1998). Rapid turnover of connexin43 in the adult rat heart. Circ. Res..

[47] Hung A.Y., Sheng M. (2002). PDZ domains: Structural modules for protein complex assembly. J. Biol. Chem..

[48] Steinhoff I., Leykauf K., Bleyl U., Dürst M., Alonso A. (2006). Phosphorylation of the gap junction protein Connexin43 in CIN III lesions and cervical carcinomas. Cancer Lett..

