# Supplementary material for: HPV16 E6 Controls the Gap Junction Protein Cx43 in Cervical Tumour Cells
Source: Viruses. 2015 Oct 5;7(10):5243–56. doi: 10.3390/v7102871 (PMC4632379; doi:10.3390/v7102871)
Supplement: Supplementary File 1 [file viruses-07-02871-s001.zip › viruses-07-02871-supplementary/supplementary Figure 1.pptx]

## Slide 1
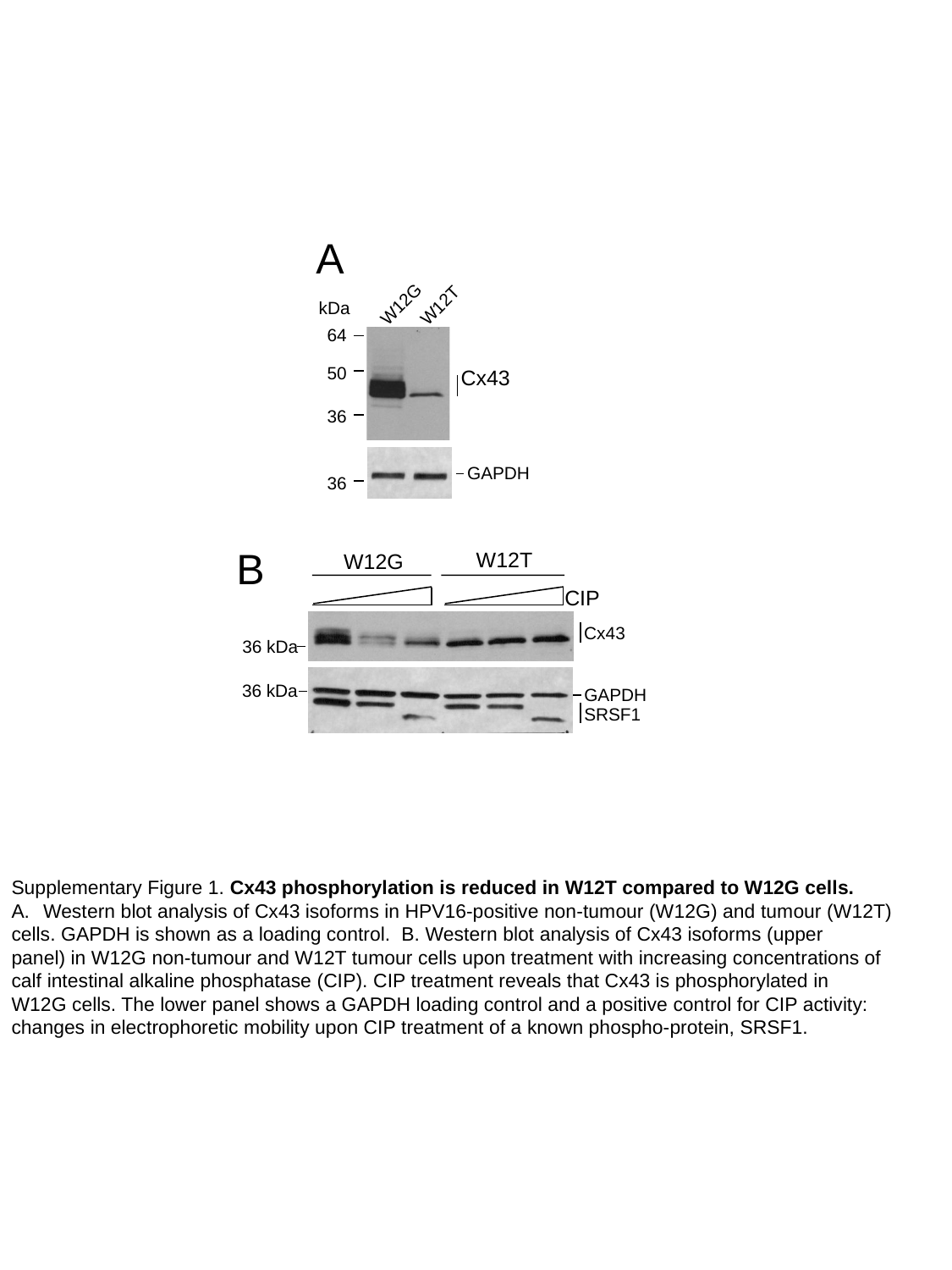

A
W12G
W12T
kDa
64
50
Cx43
36
GAPDH
36
B
W12T
W12G
CIP
Cx43
36 kDa
36 kDa
GAPDH
SRSF1
Supplementary Figure 1. Cx43 phosphorylation is reduced in W12T compared to W12G cells.
Western blot analysis of Cx43 isoforms in HPV16-positive non-tumour (W12G) and tumour (W12T)
cells. GAPDH is shown as a loading control. B. Western blot analysis of Cx43 isoforms (upper
panel) in W12G non-tumour and W12T tumour cells upon treatment with increasing concentrations of
calf intestinal alkaline phosphatase (CIP). CIP treatment reveals that Cx43 is phosphorylated in
W12G cells. The lower panel shows a GAPDH loading control and a positive control for CIP activity:
changes in electrophoretic mobility upon CIP treatment of a known phospho-protein, SRSF1.
