# Supplementary figures and images for: HPV16 E6 Controls the Gap Junction Protein Cx43 in Cervical Tumour Cells
Source: Viruses. 2015 Oct 5;7(10):5243–56. doi: 10.3390/v7102871 (PMC4632379; doi:10.3390/v7102871)

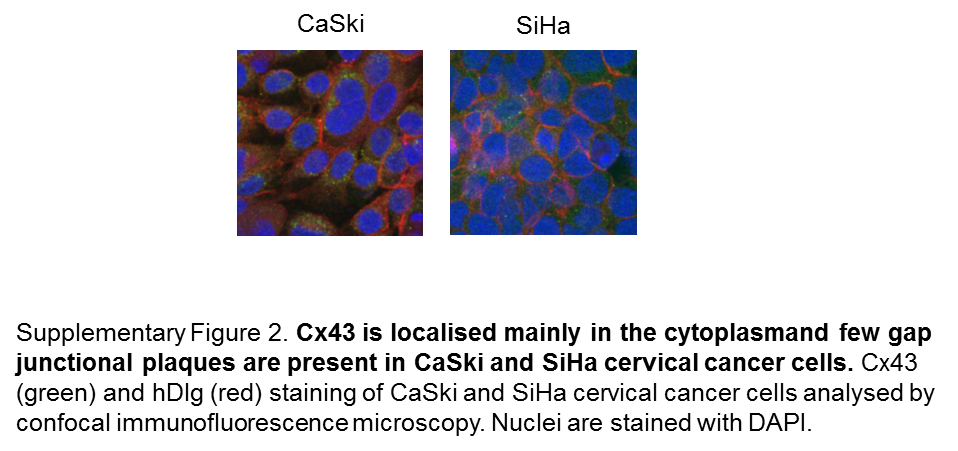

Supplement: Supplementary File 1 [file viruses-07-02871-s001.zip › viruses-07-02871-supplementary/supplementary Figure 2.png]
